# Australian Clients' Perspectives on Accessing Alcohol and Other Drug Counselling via Telehealth: A Qualitative Study

**DOI:** 10.1111/dar.70129

**Published:** 2026-02-19

**Authors:** Ashlea Bartram, Md Abdul Ahad, Dan I. Lubman, April Long, Ele Morrison, Jill Rundle, Nicole Lee, Scott Wilson, Jacqueline Bowden

**Affiliations:** ^1^ National Centre for Education and Training on Addiction, Flinders Health and Medical Research Institute Flinders University Adelaide Australia; ^2^ College of Medicine and Public Health, Flinders Health and Medical Research Institute Flinders University Adelaide Australia; ^3^ Monash Addiction Research Centre and Eastern Health Clinical School, Faculty of Medicine, Nursing and Health Sciences Monash University Melbourne Australia; ^4^ Turning Point Eastern Health Melbourne Australia; ^5^ Smart Recovery Australia Sydney Australia; ^6^ Australian Injecting and Illicit Drug Users League Melbourne Australia; ^7^ Western Australian Network of Alcohol and Other Drug Agencies Perth Australia; ^8^ Hello Sunday Morning Sydney Australia; ^9^ National Drug Research Institute Curtin University Perth Australia; ^10^ Aboriginal Drug and Alcohol Council Adelaide Australia

**Keywords:** Australia, clients, counselling, substance‐related disorders, telemedicine

## Abstract

**Introduction:**

Telehealth—the delivery of services via phone or video—has the potential to overcome barriers to attending alcohol and other drug (AOD) counselling by removing the need to physically attend appointments. However, it may raise other barriers related to technical difficulties, privacy concerns, or challenges developing a therapeutic relationship. Little is known regarding client preferences when both face‐to‐face and telehealth services are available. This study aimed to explore clients' views on the benefits and drawbacks of accessing AOD counselling via telehealth in comparison to face‐to‐face.

**Methods:**

This qualitative study involved semi‐structured interviews with 22 Australians who had received AOD counselling services via telehealth and face‐to‐face within the past 12 months. Data were analysed using an inductive thematic approach.

**Results:**

All participants indicated they would be open to accessing AOD counselling via telehealth again in the future in at least some circumstances, although their preferences for telehealth or face‐to‐face sessions varied. Participants' discussion of preferences reflected five themes: (i) telehealth helps to overcome barriers to accessing counselling; (ii) telehealth can increase confidence to engage in counselling; (iii) telehealth can limit rapport; (iv) privacy concerns with telehealth counselling; and (v) increasing awareness of telehealth can support client choice.

**Discussions and Conclusions:**

Participants viewed availability and awareness of the option to access AOD counselling via telehealth as important to client‐centred care. Service providers may need to invest in clinician training and systems development to ensure that they can provide safe, secure and effective counselling via telehealth.

## 1 Introduction

There is a substantial level of unmet need for alcohol and other drug (AOD) treatment services internationally [1]. In Australia, the setting of this study, estimates indicate that only 30%–48% of the people who would benefit from AOD treatment are able to access it [2]. A wide range of psychosocial, economic and structural barriers can inhibit engagement with AOD treatment services, including shame and stigma, affordability, long wait times and challenges in attending a service due to transport or time constraints [3, 4].

Telehealth—defined in this paper as the delivery of a treatment service by a service provider who is located remotely from a client, for example via phone or video call—may provide one approach to addressing these barriers to treatment access by removing the need to physically attend an appointment [5]. There has been a longstanding interest in the use of telehealth services for AOD treatment, with particular emphasis on delivering counselling and psychosocial support services (e.g., [6]), but the availability of telehealth services increased substantially following the advent of the COVID‐19 pandemic [7, 8, 9]. Of note, this study has primary focus on telehealth counselling and does not include telehealth for the provision of pharmacotherapy scripts in isolation. Standalone telehealth counselling services have been shown to be effective in reducing substance use problem severity [10]. Research directly comparing outcomes from telehealth and face‐to‐face AOD treatment services is currently limited, but systematic reviews from related fields such as mental health have concluded that outcomes appear to be similar for both telehealth and face‐to‐face services [11, 12]. In addition, there is evidence that telehealth may reach different cohorts of clients who are less likely to seek help from face‐to‐face services [13, 14] and may help to improve attendance rates, particularly when delivered over phone [15].

This evidence regarding reach and effectiveness is promising, but it is also important to ensure that a telehealth service meets the needs and preferences of the clients who will be using it. A limited body of recent research has examined AOD client perspectives of telehealth, primarily in the context of the COVID‐19 pandemic. Qualitative research with Australians who accessed AOD treatment via telehealth during the COVID‐19 pandemic found that participants valued telehealth as a means to access empathic care despite restrictions on face‐to‐face interaction [16]. However, some participants raised concerns about the effects of telehealth on the therapeutic alliance, while contextual factors such as location and personal resources affected their ability to access telehealth privately and reliably [16]—corroborating concerns raised by service providers that telehealth could exacerbate a digital divide [9, 17]. Many clients expressed a preference for face‐to‐face or hybrid models of delivery, in which they could choose between a combination of telehealth and face‐to‐face services [16]. A survey of telehealth clients of an outpatient substance use disorder treatment program in Massachusetts, United States during the COVID‐19 pandemic found that most clients expressed satisfaction with the quality of telehealth care, valuing the ability to attend from home and avoid travel time [18]. Reasons for disliking telehealth included the ability for telehealth to be interrupted by others as well as challenges connecting with people via telehealth (particularly for group therapy) [18]. Outside of the COVID‐19 pandemic context, an interview study with clients accessing a standalone Australian telephone counselling service for mild‐to‐moderate alcohol use disorder highlighted that telehealth could overcome barriers to accessing treatment by providing a greater sense of anonymity as well as access outside of traditional hours [19]. However, similar to previous studies, some participants perceived the lack of face‐to‐face interaction as an impediment to developing rapport with their counsellor [19].

This existing research suggests that telehealth may be of value to at least some AOD clients. However, this research has been undertaken in contexts in which there was limited or no availability of face‐to‐face services, reducing the extent to which participants could make an informed comparison between the two delivery modes. Increasingly, both face‐to‐face and telehealth services may be available to clients, with clients having the option to move between delivery modes [20]. In this study, we sought the perspectives of people who have accessed AOD counselling via both telehealth and face‐to‐face, aiming to develop an understanding of their views on the benefits and drawbacks of accessing AOD counselling via telehealth based on their experiences of counselling via both delivery modes.

## 2 Methods

### 2.1 Study Design

We employed a qualitative interview study design under a realist/essentialist epistemological framework, viewing the aim of the research as understanding and reporting the experiences, meaning and reality of participants with lived experience of receiving AOD counselling via telehealth and face‐to‐face [21].

### 2.2 Participants and Procedure

Participants of this study were 22 people living in Australia aged 18 years and above who had received AOD counselling via telehealth and face‐to‐face within the past 12 months. Participants were recruited via convenience sampling between August and November 2024: a call for participants was shared via AOD treatment organisations that had participated in previous research regarding their use of telehealth and expressed a willingness to be contacted regarding other projects, AOD peak representative bodies and the research team's social media accounts.

Interested participants were provided with study information and a copy of the interview schedule and invited to take part in a single 30‐min interview with AB, an experienced female mixed‐methods researcher, via Microsoft Teams or phone, depending on each participant's preference. To the interviewer's knowledge, no non‐participants were present during interviews. Participants either provided written consent prior to the interview or recorded verbal consent at the beginning of their interview. Interviews were semi‐structured, beginning with a broad invitation for participants to describe their experiences with accessing AOD counselling via phone or video. Follow‐up prompts relating to choice of delivery mode, privacy, preparation, communication, satisfaction, barriers and facilitators to accessing phone or video counselling, and future intentions to use phone or video, as well as basic demographic information. Across the interview, participants were encouraged to make comparisons with their experiences of face‐to‐face counselling. The interview schedule was developed with input from the full research team, which included people with lived/living experience of AOD, but was not formally pilot tested.

There was no prior relationship between the interviewer and any of the participants. At interview commencement, participants were advised that the interview would focus on processes rather than the content of their counselling sessions, and that they could choose not to answer any question. Interview durations ranged from 16 to 46 min, with an average length of 25.5 min. Each participant was provided with an AU$30 gift voucher for their participation in the study.

Interviews were recorded and automatically transcribed in Microsoft Teams, with transcripts de‐identified and reviewed for accuracy by AB and MAA. Participants were provided with the option to review their transcripts and request contents be excluded from analysis; 19 participants elected to receive their transcript, but none requested that any data be excluded.

### 2.3 Data Analysis

The reviewed transcripts were imported into NVivo 12 software and analysed using an inductive thematic approach as per the methodological guidelines of Braun and Clarke [21]. MAA read and re‐read transcripts to familiarise himself with the data, then inductively coded each transcript. MAA and AB refined and sorted the codes into multiple hierarchical structures indicating sub‐themes and then developed preliminary themes by collating related sub‐themes. Preliminary themes were then reviewed by all authors before being finalised.

### 2.4 Reflexivity

The authorship team comprised people with diverse backgrounds in the AOD field, including research, service design and administration (including the design and administration of telehealth services), clinical practice, policy development, advocacy, and lived and living experience of AOD and service use. All authors have previously collaborated on a paper examining addiction treatment via telehealth from the service providers' perspectives [20]. A.B. and M.A.A., as lead analysts, are currently employed as researchers. They have PhDs and extensive experience in conducting qualitative interview research but no background in clinical or counselling practice. Reflexive discussions with the broader authorship team helped to maintain awareness of assumptions and ensure that identified themes were grounded in participants' accounts of their experiences and the broader AOD counselling literature.

### 2.5 Ethical Approval

This study had ethical approval from Flinders University's Human Research Ethics Committee (project number 7484).

## 3 Results

### 3.1 Demographic Characteristics

Table 1 shows the demographic characteristics of participants. Eleven participants were male (50%), nine were female (41%) and two identified as non‐binary. Participants' ages ranged from 22 to 65. Participants most commonly described their ethnicity as African (32%), Caucasian/Anglo‐Australian (23%) and/or Black (23%). Eighteen participants (82%) were from capital cities and four (18%) were from regional Australia.

**TABLE 1:** Demographic characteristics of participants (*N* = 22).

Demographic traits	*N*	%
Sex		
Male	11	50
Female	9	41
Non‐binary	2	9
Age, years		
20–29	8	36
30–39	9	41
40 and above	5	23
Education		
High school	10	45
Undergraduate	9	41
Postgraduate	3	14
Employment		
Employed	14	64
Not employed	8	36
Ethnicity^a^		
African	7	32
Anglo‐Australian/Caucasian	5	23
Black	5	23
Aboriginal and/or Torres Strait Islander	2	9
Other	5	23
State		
Victoria	7	32
New South Wales	6	27
South Australia	5	23
Queensland	3	14
Western Australia	1	5
Area of residence		
Capital city	18	82
Regional	4	18

^a^ Participants could nominate more than one ethnicity, so numbers do not sum to 22.

### 3.2 Clients' Experiences of Telehealth Delivery by Australian Counselling Services

Overall, all participants indicated that they would be willing to access AOD counselling via telehealth again in the future in at least some circumstances, although participants varied in the extent to which they expressed a preference for telehealth or face‐to‐face counselling. Participants articulated a range of benefits, disadvantages and additional considerations regarding the use of telehealth for AOD counselling, which have been organised into five overarching themes: (i) telehealth can help overcome structural barriers; (ii) telehealth can increase confidence to engage in counselling; (iii) telehealth can limit the development of rapport; (iv) privacy concerns with telehealth counselling; and (v) increasing awareness of telehealth can support client choice.

### 3.3 Theme 1: Telehealth Helps Overcome Structural Barriers to Accessing Face‐To‐Face Counselling

Participants viewed telehealth as something that was easily accessible, significantly reducing structural barriers to accessing counselling services for AOD treatment. In general, participants said telehealth counselling offered more flexibility and time efficiency when it came to scheduling appointments to fit within their other plans and responsibilities, as compared to face‐to‐face counselling.

The reason why I have started having my counselling sessions with her over the phone, it is just the convenience because I work as a carer, I also look after my mum, I'm my mum's full time carer on top of having an occupation as a carer so mainly it has a lot to do with time‐effectiveness, the lack of time I have all the time basically, so to drive to [suburb] and back from where I live is about half an hour there and back. So that'll be an hour driving. (P. 16, Metropolitan Adelaide, Female)

The use of telehealth counselling also enabled easier access to counselling for participants who experienced health or mobility challenges or who lived in regional areas. For example, one participant who lived in a regional area noted:

I guess for me it was tricky to sort of engage in any sort of counselling being where I live in a sort of a large rural city […] I was not driving at the time, I didn't have a license back yet, so transport was a bit of an issue, it would take me an hour and a half to get somewhere for an appointment where I could just log in online. So I guess the ease at which I could have the appointments. (P. 13, Regional Victoria, Female)

Most participants believed that telehealth services reduced the time and cost associated with physically attending alcohol and drug counselling by avoiding the need to travel and reducing the time spent in waiting rooms.

But if I was at home, I could be doing something because we have a time. If it's 6:00 PM, so I could do whatever I wanna do before 6:00 PM, and that's fine. Whenever I'm at her office, I have to wait some time for if she maybe had another stretch with someone. So I think that online gives your time, gives you time like it, it takes care of your time a lot, yeah. […..] Apart from the service fee and all that, but when you go to the office, you have to, you know, buy [petrol, parking for] your car and then move down there and all of that inconvenience. (P. 10, Metropolitan Brisbane, Female)

Despite the fact that telehealth was viewed as overcoming the burden of attending face‐to‐face counselling sessions, participants acknowledged that technical difficulties could present their own barrier. For example:

I was having a really poor connection […] Face‐to‐face, that problem is not even a problem because it can't happen [….] It was over 20 min of, you know, could we connect it, disconnect and all. (P. 9, Metropolitan Melbourne, Male)

Participants reported interruptions resulting from limited or unstable internet access in certain areas, device malfunctions and/or low levels of digital literacy. However, some participants also noted that their service providers worked with them to address these interruptions, for example by switching from video to phone in ‘times where maybe my bandwidth wasn't that great’ (P. 18), or by providing technical support to help them to use the platform:

I was given a list of things to do and I checked all that before […] I had to use my computer, I was quite used to you know having a video call on my phone so it was kind of the first time I was using it on my computer. So it was a little bit shaky. (P. 20, Regional Queensland, Transgender Man)

### 3.4 Theme 2: Telehealth Increases Clients' Confidence to Engage in Counselling

Many participants described telehealth as something that increased their confidence to engage in AOD counselling. They viewed telehealth as enabling them to engage from the comfort of their own home, reducing their fear of stigma and limiting social anxiety. Participants described an increased confidence in engaging openly with their counsellor via telehealth, facilitated by a sense of relief in being able to speak about deeply personal and potentially stigmatised experiences which supported them to build a therapeutic alliance:

I think it gives me more confidence [….] I can actually speak about my worries about how close I was to taking on that shot. How close I was to taking out the stick. (P. 8, Metropolitan Melbourne, Male)

Yes, in a space of, in a sense of safety and connection, and you know, so it helps to build a stronger therapeutic relationship. (P. 3, Metropolitan Melbourne, Male)

Clients reported being more relaxed in their own private environment, finding it a calmer experience than attending a clinical setting:

The sessions actually provide a comfortable and a private environment. So, I just saw that as something I could actually benefit from because it could keep me at a very calm place to you know, be able to access the service easily. (P. 2, Metropolitan Brisbane, Male)

Several participants also noted that accessing counselling via telehealth allowed them to more easily manage feelings of shame and social anxiety:

So I'm a fairly introverted person […] I also get very nervous around people. You know, I get very highly sensitive. So even and especially opening up to even someone that I'm comfortable with, even that can cause a lot of mental and emotional stress to me. So I think that's one of the main reasons I still want to go with phone. (P. 16, Metropolitan Adelaide, Female)

We do isolate and we have shame and, you know, it is hard for us to get out of the house. […] I shouldn't say us, it was hard for me to get out of the house so. You know, for whatever reasons it was so, yeah, just knowing that [telehealth's] an option. (P. 22, Regional South Australia, Female)

In addition, for clients who accessed counselling via video, having permission to turn off the camera when needed enhanced their sense of control over the process:

My thinking about either turning off my camera, turning it on was I felt I had all the options at my hands, at my disposal so I felt OK. […] I could pick whatever I'm comfortable with. (P. 7, Metropolitan Sydney, Male)

Several participants noted that telehealth allowed them to avoid the discomfort or stress of sitting in front of strangers. Even if they felt comfortable meeting their counsellor in person, participants described feeling stigmatised by others they encountered in the process of accessing face‐to‐face counselling, including in waiting rooms, which may contribute to a sense of unease and marginalisation. Hence, they preferred telehealth which was found to be more emotionally safe. For one participant, he attributed the stigma to racism:

With the face‐to‐face experience, you have to wait [in the waiting room] for the scheduled time. So people around, I don't know how to explain it, the gestures I was getting from people around wasn't at all [nice]. I felt okay, maybe because race is the reason I'm getting this. So [telehealth] is something I prefer much more than face‐to‐face. (P. 12, Metropolitan Sydney, Male)

By attending counselling remotely, he was able to avoid stigmatised reactions from other people, interacting only with his counsellor with whom he felt safe.

### 3.5 Theme 3: Telehealth Can Limit the Development of Rapport Compared to Face‐To‐Face Counselling

Due to the physical separation of client and counsellor in telehealth sessions, many participants believed that the level of human interaction was reduced in comparison to face‐to‐face counselling. Some clients expressed difficulty building strong rapport, with some feeling like they had not received the same level of care when undergoing phone or video counselling for their AOD issues compared to their experiences with face‐to‐face counselling.

I think, again, if you're really struggling, as in you know, really needing that personal contact, I mean a lot of counsellors don't do like, you know, hugs and stuff like that. And I get those boundaries and things like that. But I think even sitting in a room with someone, sometimes, you know. [….]. That's personally anyway, you know sometimes over the phone you just don't feel like you're getting that connection, that you really need, and you're getting that support that you really need. (P. 19, Metropolitan Adelaide, Female)

In their opinion, telehealth presented a barrier in adequately understanding communication nuances like facial or bodily cues that would have been possible through in‐person counselling.

It's yeah, telehealth is OK. But I find the face‐to‐face interactions much more effective. I think that she can, that she's very good at reading my body language and she's absolutely, obviously, trauma informed and specialises in women that have been through trauma, and I think that our body definitely speaks a lot as humans in general, certainly women that have been through trauma. (P. 22, Regional South Australia, Female)

Some participants reported that their counselling service provided a professional image and biography of their counsellor prior to their initial counselling session to enhance familiarity and reduce a sense of impersonality in the therapeutic encounter:

I was sent the background of the counsellor I was going to have this conversation with, and the name and also the photo attached to it. […] And so you feel comfortable, ready before you have the interview with the counsellor. (P. 15, Metropolitan Melbourne, Male)

Other participants were able to increase their sense of connection with their counsellor by incorporating some initial face‐to‐face sessions into their treatment, then continuing via telehealth or a mixture of modes:

And then I explained to them that I had difficulty opening up unless I see them face‐to‐face at least once because I had this phobia of not knowing the person on the end of the phone […]. And once I've made my personal contact, I didn't hesitate to have phone calls or they'd come to my home or I'd go to their office. (P. 17, Metropolitan Adelaide, Female)

A few participants actively valued the reduced level of human interaction over telehealth, as they found it supported them to concentrate deeply on their own feelings and experiences rather than feeling pressured to perform social niceties or engaging in the cognitive and emotional effort of interpreting non‐verbal cues during their counselling session:

I think one of the main reasons I find it to be of more benefit over the phone than face‐to‐face is because I, when we are with people in therapy, like in the same room close to each other, you're picking up each other's energies on a physical level, reading each other's body language, having to make eye contact to seem normal, processing each other's body language. That is something that we don't have to do when we have counselling over the telephone. (P. 16, Metropolitan Adelaide, Female)

### 3.6 Theme 4: Privacy Concerns With Telehealth Counselling

Many participants acknowledged that they had initially held concerns regarding privacy and confidentiality when using telehealth to access AOD counselling. However, participants reported that they were willing to trust their counsellors' reassurances regarding the security and safety of their information:

Yeah, I had that concern at first, but I asked, I was open and they were open at their own end and I was assured that it's confidential, just like how you explained this to me much earlier [as part of the informed consent process]. I was asking because you may not be secured and all. So yeah, did have concerns, but it was thrashed out. (P. 20, Regional Queensland, Transgender Man)

Participants acknowledged that concerns about privacy and security of data might discourage some people from accessing telehealth. For example, the possibility of someone recording them and disclosing it to others without consent, or scammers hacking a system, had occurred to some participants:

You kinda can't hide your voice […] so you know maybe sometimes you can find someone […] Not everyone will accept [telehealth] because in the first place we do believe there maybe like the scammers, maybe your information will go out or something. (P. 5, Metropolitan Melbourne, Female)

However, other participants held very little concern, viewing their information as unlikely to be of interest or value to others:

It's not like I'm caught up with any information that would be of a concern to release for law enforcers or hackers, there's nothing that's really, my conversations are too domestic, it's too personalised about things. (P. 16, Metropolitan Adelaide, Female)

Several participants noted that breaches in privacy were more likely to occur due to their home environment, rather than the security of the systems used by services:

So if you're in a large family or you've got lots of kids at home or you're in share accommodation or something like that, makes it difficult to get that privacy. (P. 6, Metropolitan, Male)

A few participants, however, viewed telehealth as offering more privacy than face‐to‐face counselling, because it permitted them a greater degree of anonymity. For example, one participant explained that, despite a general preference for face‐to‐face counselling, she might seek phone counselling where she could avoid identifying herself out of a concern that a counsellor might have to make a child protection report:

If I was really close to a relapse, I might prefer not to have a face‐to‐face call or contact my AOD counsellor. I might just want to call someone on the phone […] This is, I suppose, a fear from what I've experienced in the past in regards to my children being taken, and it would be a personal fear, it would be nothing personal towards the worker or the agency, but like I just, that would be a conversation I'd want to have with someone who probably wouldn't know enough about me to make a report to [the local child protection agency]. (P. 13, Regional Victoria, Female)

### 3.7 Theme 5: Increased Awareness of Telehealth Would Support Client Choice

Participants firmly agreed that telehealth should be available as an option for people to access AOD counselling. However, many noted that they had limited awareness that this was an option, with participants recounting how they first encountered the possibility of telehealth late in the booking process or after they had already commenced face‐to‐face counselling:

When I was putting up for counselling, I didn't have any idea that it could be done over the phone or video, so it was last minute. (P. 15, Metropolitan Melbourne, Male)

So at the end of every appointment I've had with [counsellor], for the face‐to‐face ones and over the phone ones, one of the last questions will be how do you want to do the next session? Do you want to do face‐to‐face or over the phone? So I only really discovered after my first face‐to‐face session that she offered over the phone. (P. 16, Metropolitan Adelaide, Female)

To support clients to engage in AOD counselling, many participants believed there needed to be greater awareness that telehealth counselling services were available, for example by making this option more prominent on services' websites, booking systems or other communications. For the following participant, increasing awareness of telehealth as an option would have the potential to encourage those struggling with the confidence to attend a face‐to‐face service to nonetheless seek help:

People knowing that [telehealth] is available as opposed to face‐to‐face. [….] So just know that that's an option would be the first thing that comes to my mind is that I think a lot of people think about counselling as face‐to‐face as opposed to there being other options. (P. 22, Regional South Australia, Female)

For some participants, in addition to raising awareness of telehealth and AOD counselling per se, they also saw a need to increase awareness of the measures that services employed to protect clients' privacy over telehealth. Although most had been reassured about these measures by their counsellor or the service as part of the booking process, they felt that making this information more prominent in advance might stop others with stronger privacy concerns from being deterred:

I think having that assurance that [you can trust that your data will be secure], if that would be laid out, for me, that would be huge in boosting people's morale to engage in phone counselling […] I think having it on public places like the website [would help]. (P. 21, Regional New South Wales, Non‐binary)

## 4 Discussion

This study sought the perspectives of clients on the benefits and drawbacks of accessing AOD counselling via telehealth compared to face‐to‐face. All clients saw a role for telehealth on at least some occasions, despite varying in their individual preferences for each delivery mode. Access to telehealth—and the promotion of this access—was viewed as an important part of providing clients with choice regarding their treatment, so that it was responsive to their needs and preferences. This is a noteworthy finding given that client‐centred and flexible approaches have been identified as central to facilitating AOD treatment attendance [22].

For some participants in this study, telehealth was valued primarily for its ease of accessibility, with participants expressing a willingness to trade off what they perceived as a somewhat inferior counselling experience for benefits such as reduced travel and waiting times, lower costs and increased flexibility to access the service around their other life commitments. This sentiment echoes findings from studies conducted in the context of the COVID‐19 pandemic, where participants valued the ability to attend services from home in the face of restrictions on face‐to‐face interactions, while benefiting from reduced travel time and enabling more efficient use of time—particularly by avoiding lengthy waiting times in clinical settings [16, 18]. Additionally, standalone AOD telehealth treatment services have been found to significantly reduce transport and indirect costs when compared to face‐to‐face treatment [23]. Use of telehealth to access AOD treatment has been associated with increased engagement and retention, at least in the context of COVID‐19 [8, 24]. AOD counselling service providers have identified accessibility as a key benefit of telehealth, particularly for under‐served populations [20]. However, they also expressed concerns over the extent to which they could develop effective therapeutic relationships via phone or video [20].

In contrast to this concern from service providers, some participants in this study actively preferred to access counselling via telehealth regardless of the accessibility of face‐to‐face, finding telehealth to provide as or more satisfying an experience than face‐to‐face counselling. Clients attributed this experience to being able to attend sessions in a comfortable, familiar environment, leading them to feel less anxious, shamed and stigmatised than when attending sessions in person. Some clients also valued the greater sense of anonymity and reduction in visual cues, discovering that this reduced cognitive and emotional demands and enabled them to discuss their feelings and experiences more easily. Findings from this and other studies suggest that many clients are able to develop trusting therapeutic alliances with their counsellor via telehealth [25, 26]. Previous research has identified that clinicians' and clients' perspectives of the therapeutic alliance do not always match [27], with the latter being more relevant to clinical outcomes [28]—underscoring the importance of seeking client perspectives.

A limitation of this study was its use of a convenience sample that was not representative of all clients who access or might benefit from accessing AOD counselling. The conditions of our ethics approval did not allow us to collect details regarding participants' substances of concern or past treatment experiences, although self‐disclosures from some participants enabled us to infer that the sample included a mix of participants who had sought treatment for either alcohol or other drugs, and a mix of participants with either some or no prior history of seeking AOD treatment before their use of telehealth. We recruited only a small number of participants who lived outside of capital cities. People from regional and remote areas can face greater barriers to accessing AOD counselling, including both structural barriers such as the distance to the nearest available service as well as increased concerns around stigma due to perceived challenges to accessing services anonymously in smaller towns [3]. As such, telehealth may be of particular interest for this cohort. This study's focus on participants with experience of both telehealth and face‐to‐face AOD counselling, while providing greater insight into the comparative benefits and drawbacks of each mode, will have excluded the perspectives of people who were only able or willing to access AOD counselling via telehealth—potentially including those from regional areas with limited access to face‐to‐face services. Future studies could directly explore the perspectives of people who seek AOD treatment from regional areas or other priority or underserved populations on the role of telehealth in AOD counselling. In addition, future research should also investigate the number of sessions an individual was involved in and how this may have been related to their experience of counselling. There may also be value in exploring the perspectives of people who struggle to engage with both face‐to‐face and telehealth services, to further understand the limitations of each mode and identify alternative approaches to meeting these people's treatment needs.

### 4.1 Implications for Policy and Practice

This study's findings suggest that providing options to access AOD counselling via telehealth aligns with client preferences and may reduce barriers to treatment access. Noting that participants varied in the extent to which they felt they could develop a therapeutic alliance with their counsellor remotely, there may be a need for services to upskill clinicians in effective delivery of counselling over phone and video. Organisations such as the British Association for Counselling and Psychotherapy have developed competence frameworks and curricula to support training and practice in phone and video counselling [29]. Supporting counsellors to undertake professional development on this topic may help to reduce the sense expressed by some (but not all) participants of making a trade‐off between accessibility and effectiveness. Counsellors should also seek feedback from clients about their experiences of accessing counselling via telehealth and their sense of therapeutic alliance. Services could consider options for hybrid delivery that allow participants to move between face‐to‐face and telehealth to suit their needs and preferences. Services should also attend to the security of their telehealth platforms and processes to ensure that participants' trust in counsellors is not misplaced and clearly communicate to prospective clients if and how telehealth services can be accessed to maximise the potential for this mode to address unmet need.

## 5 Conclusion

Although clients saw both benefits and drawbacks to accessing AOD counselling via telehealth, they indicated that having telehealth available as an option for AOD counselling was important to support client‐centred care. Despite on occasion encountering new (technological) barriers, participants saw great value in being able to access AOD counselling flexibly with the support of counsellors who could maintain effective therapeutic spaces via telehealth.

## Author Contributions

Each author certifies that their contribution to this work meets the standards of the International Committee of Medical Journal Editors.

## Funding

This work was supported by the Department of Health and Aged Care, Australian Government (4‐IN4X1RZ).

## Conflicts of Interest

The authors declare no conflicts of interest.

## Data Availability

The data are not available due to privacy or ethical restrictions.
